# Hydrogen Sulfide Lowers Proliferation and Induces Protective Autophagy in Colon Epithelial Cells

**DOI:** 10.1371/journal.pone.0037572

**Published:** 2012-05-29

**Authors:** Ya C. Wu, Xiao J. Wang, Le Yu, Francis K. L. Chan, Alfred S. L. Cheng, Jun Yu, Joseph J. Y. Sung, William K. K. Wu, Chi H. Cho

**Affiliations:** 1 School of Biomedical Sciences, The Chinese University of Hong Kong, Shatin, NT, Hong Kong; 2 Institute of Digestive Disease, LKS Institute of Health Sciences and Department of Medicine & Therapeutics, The Chinese University of Hong Kong, Shatin, NT, Hong Kong; Virginia Commonwealth University, United States of America

## Abstract

Hydrogen sulfide (H_2_S) is a gaseous bacterial metabolite that reaches high levels in the large intestine. In the present study, the effect of H_2_S on the proliferation of normal and cancerous colon epithelial cells was investigated. An immortalized colon epithelial cell line (YAMC) and a panel of colon cancer cell lines (HT-29, SW1116, HCT116) were exposed to H_2_S at concentrations similar to those found in the human colon. H_2_S inhibited normal and cancerous colon epithelial cell proliferation as measured by MTT assay. The anti-mitogenic effect of H_2_S was accompanied by G_1_-phase cell cycle arrest and the induction of the cyclin-dependent kinase inhibitor p21^Cip^. Moreover, exposure to H_2_S led to features characteristic of autophagy, including increased formation of LC3B^+^ autophagic vacuoles and acidic vesicular organelles as determined by immunofluorescence and acridine orange staining, respectively. Abolition of autophagy by RNA interference targeting Vps34 or Atg7 enhanced the anti-proliferative effect of H_2_S. Further mechanistic investigation revealed that H_2_S stimulated the phosphorylation of AMP-activated protein kinase (AMPK) and inhibited the phosphorylation of mammalian target of rapamycin (mTOR) and S6 kinase. Inhibition of AMPK significantly reversed H_2_S-induced autophagy and inhibition of cell proliferation. Collectively, we demonstrate that H_2_S inhibits colon epithelial cell proliferation and induces protective autophagy via the AMPK pathway.

## Introduction

Hydrogen sulfide (H_2_S) is produced by indigenous sulfate-reducing bacteria in the large intestine and may reach concentrations ranging from 0.3 to 3.4 mmol L^−1^ in human colon [1], [2], [3]. In addition, H_2_S may be synthesized endogenously from L-cysteine by cystathionine synthases. It has been reported that the amount of H_2_S is increased while the expression of H_2_S-catabolising enzymes is reduced in colon cancer patients [4], [5]. Moreover, fecal sulfide is elevated in patients with ulcerative colitis, a condition associated with an increased risk for colon cancer [6]. Whether elevation of H_2_S is the cause or the result of colon carcinogenesis and its effect on normal and cancerous colon epithelial cells, however, remains elusive.

Macroautophagy (hereafter referred to as ‘autophagy’) is an evolutionarily conserved lysosome-dependent pathway for protein degradation. Autophagy is initiated by the formation of autophagosomes which nonselectively sequester long-lived proteins and cytoplasmic organelles such as mitochondria, endoplasmic reticulum and ribosomes. Autophagosomes then fuse with acidic lysosomes to produce autolysosomes when the lysosomal hydrolases digest the engulfed contents. Free amino acids obtained return to the cytoplasm for reuse. While overactivation of autophagy is incompatible with cell growth, this self-cannibalistic process may protect cells from various kinds of stress, such as nutrient starvation, cytotoxicity of cancer therapeutics or anoikis induced by the loss of contact with the extracellular cell matrix [7], [8], [9], [10]. In relation to its regulation, the AMP-dependent kinase (AMPK) and mammalian target of rapamycin (mTOR) pathways play important roles in the control of autophagy. To this end, activation of AMPK or inhibition of mTOR function has been shown to activate autophagy [11]. In the present study, we demonstrate the induction of cell cycle arrest and protective autophagy by H_2_S in normal and cancerous colon epithelial cells and the involvement of AMPK and mTOR signaling.

## Results

### Hydrogen sulfide inhibited colon epithelial cell proliferation and colon cancer cell migration

**Figure 1:**
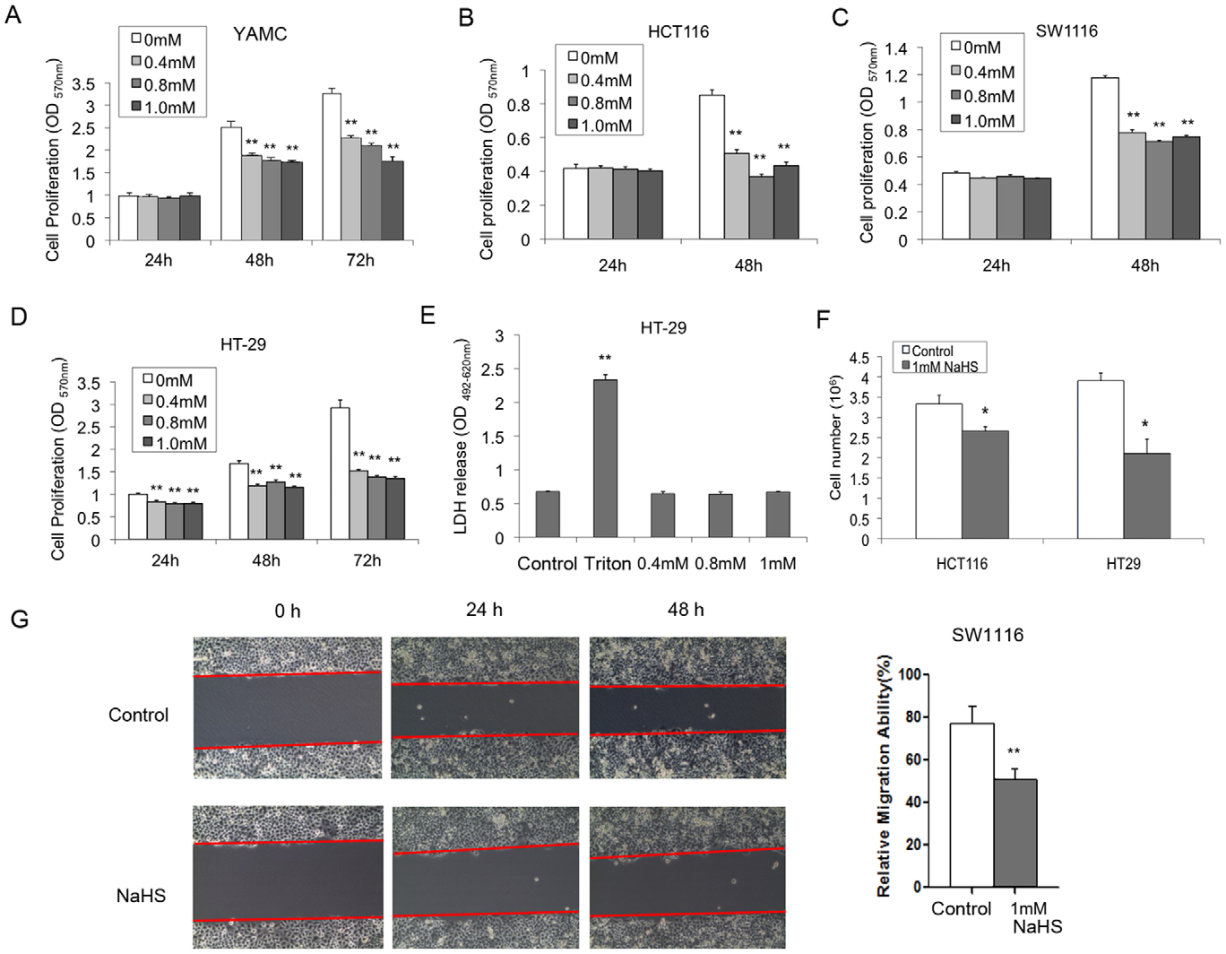
Inhibition of colon epithelial cell proliferation and migration by H_2_S. Incubation of NaHS reduced, in a dose- and time-dependent manner, cell proliferation of (A) YMAC, (B) HCT116, (C) SW1116 and (D) HT-29 as determined by MTT assay. (E) NaHS did not affect necrotic cell death in HT-29 as determined by lactate dehydrogenase release assay. 1% (v/v) Triton X-100 was used as a positive control. (F) The anti-proliferative effect of NaHS (24 h) on colon epithelial cells was further confirmed by direct cell counting. (G) NaHS (1 umol L^−1^) significantly reduced the migration of SW1116 cells as measured by wound healing assay. ^*^ *P*<0.05; ^**^ *P*<0.01, significantly different from respective control group.

To study the effect of H_2_S on proliferation of colonocytes, we examined changes in MTT tetrazolium salt formation in normal (YAMC) and cancerous (HT-29, HCT-116, SW1116) colon epithelial cell lines. As shown in **Fig. 1A–D**, H_2_S at physiological concentrations significantly reduced MTT tetrazolium salt formation in YAMC cells and all three colon cancer cell lines in a time-dependent manner. At the dose of 1 mmol L^−1^, 72-h treatment of NaHS inhibited YAMC and HT-29 cell proliferation by 45% and 50%, respectively. The anti-mitogenic effect of H_2_S could be observed as early as 24 h after treatment in HT-29 and SW1116. Necrotic cell death in HT-29 was confirmed to be unaffected by H_2_S treatment as determined by lactate dehydrogenase release assay which measured the integrity of plasma membrane (**Fig. 1E**). Moreover, there was no DNA ladder formation or aggregation of the nucleus under electron microscope after 48 h treatment in HT-29 cells (data not shown), suggesting that apoptosis could not account for the reduction of cell proliferation induced by H_2_S. To further confirm the anti-proliferative effect of H_2_S, direct cell counting of HCT1116 and HT-29 cells treated with or without H_2_S was performed. Results show that 24 h of H_2_S treatment significantly reduced cell number in both cell lines (**Fig. 1F**). To determine if H_2_S could alter cell migration, wound healing assay in the presence or absence of NaHS in SW1116 cell was performed. As shown in **Fig. 1G**, H_2_S significantly reduced SW1116 cell migration at 48 h.

### Hydrogen sulfide inhibited G_1_-S transition and induced p21^Cip1^ expression in colon epithelial cells

**Figure 2:**
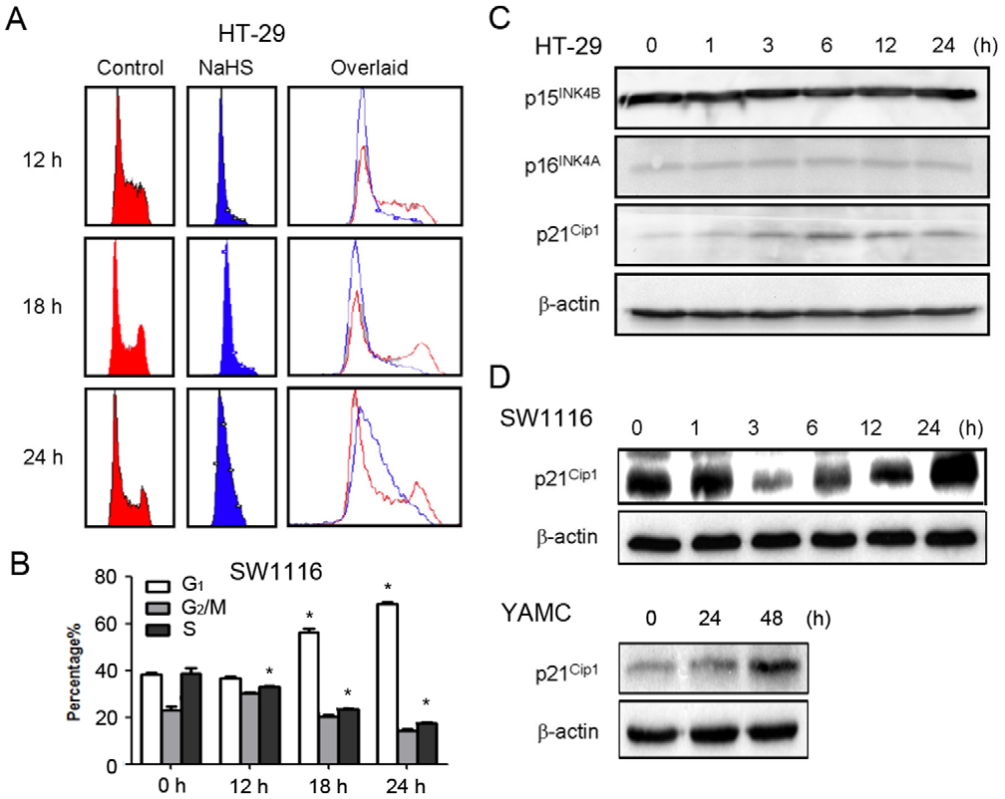
Inhibition of cell cycle progression of colon epithelial cells by H_2_S. (A) DNA histogram shows the accumulation of G_0_/G_1_-phase cells induced by NaHS in HT-29. Cells were treated without or with 1 mmol/L NaHS, and their DNA contents were determined by flow cytometry analysis. (B) Quantitative analysis of cell cycle distribution of SW1116 cells treated with or without 1 mmol/L NaHS at various time points. (C) NaHS (1 mmol/L) time-dependently up-regulated p21^Cip1^ protein expression. The induction was maximal 6 h after NaHS treatment. (D) The effect of H_2_S on p21^Cip1^ induction was confirmed in SW1116 and YAMC cells. The results are the representative of three independent experiments. ^*^ *P*<0.05, significantly different from respective control group at 0 h.

To further confirm the anti-mitogenic action of H_2_S, flow cytometry-based cell cycle analysis was performed. Results showed that 12 h- or 18 h-treatment of NaHS at the concentration of 1 mmol L^−1^ induced a substantial accumulation of HT-29 cells at the G_0_/G_1_-phase in a time-dependent manner (**Fig. 2A**). A reciprocal reduction of proportion of cells in S and G_2_/M-phases was also observed in NaHS-treated HT-29 cells. The G_1_-arresting effect of H_2_S was confirmed in SW1116 cells (**Fig. 2B**). At all time points, no increase in sub-G_1_ phase, which was indicative of apoptotic cells, was observed. The effect of H_2_S on the expression of several cell cycle regulators in colon cancer cells was determined in HT-29. Results showed that NaHS induced a time-dependent up-regulation of the cyclin-dependent kinase inhibitor p21^Cip1^. The expression of other cell cycle regulators, such as p15^Ink4b^ and p16^Ink4a^ were not affected (**Fig. 2C**). The induction of p21^Cip1^ by H_2_S was also confirmed in SW1116 and YAMC cells (**Fig. 2D**). In SW1116 cells, NaHS (1 mmol L^−1^) transiently lowered p21^Cip1^ expression at 3 h and 6 h but caused significant induction at 24 h. In YAMC cells, remarkable induction of p21^Cip1^ was observed at 48 h after treatment with NaHS (1 mmol L^−1^).

### Hydrogen sulfide induced the formation of LC3B^+^ autophagosomes and LC3-II accumulation

**Figure 3:**
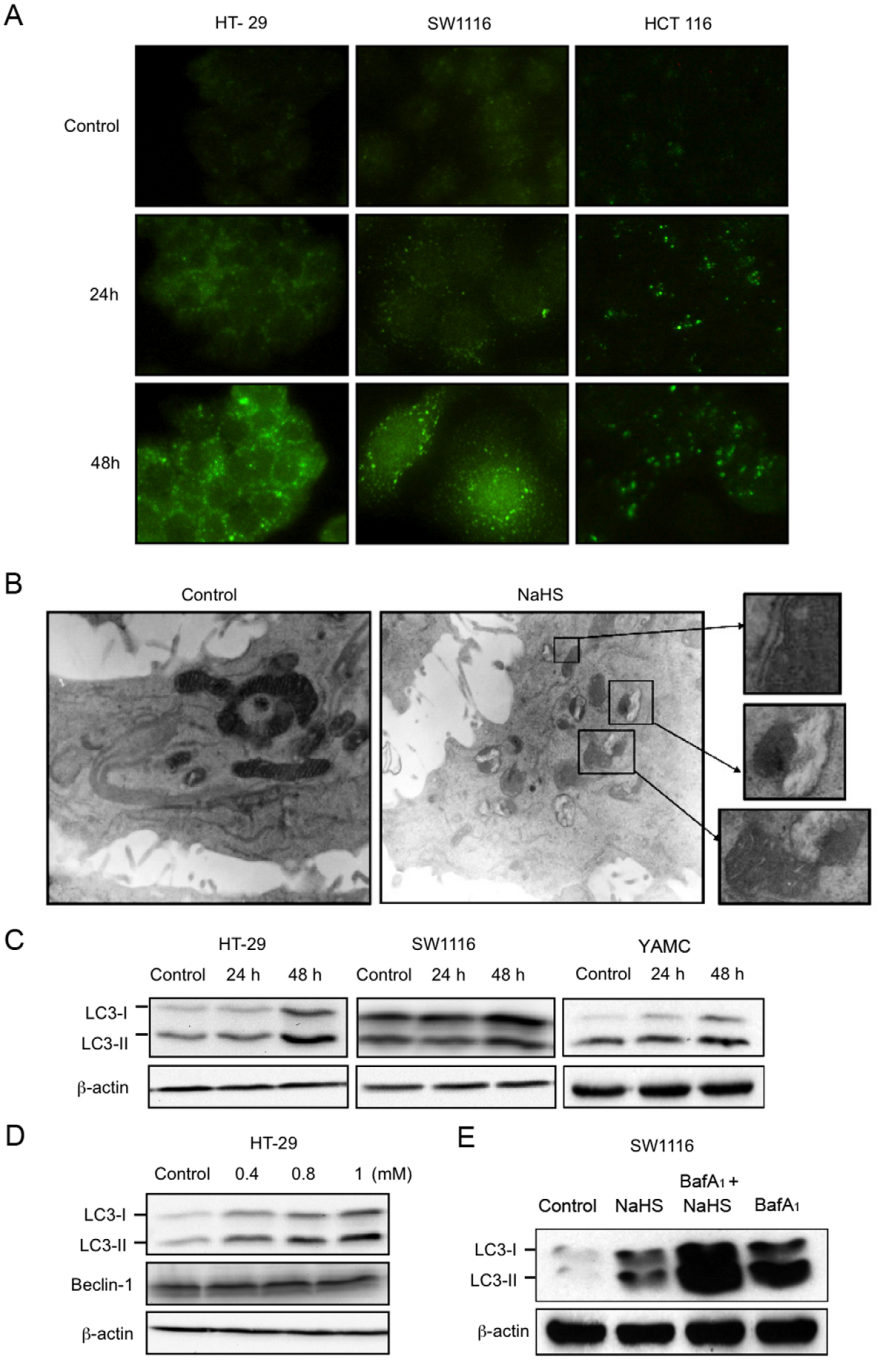
Induction of autophagosomes in colon epithelial cells by H_2_S. (A) Treating the cells with NaHS for 24 h or 48 h prominently enhanced the formation of autophagic vacuoles as determined by immunofluorescent staining for LC3B. (B) Ultrastructural analysis by electron microscopy revealed the formation of autophagosome or secondary lysosomes with the residual digested material in NaHS-treated HT-29 (1 mmol/L; 48 h). (C) NaHS increased LC3-II levels in HT-29, SW1116 and YAMC cells after treatment with NaHS (1 mmol/L) for 48 h. (D) NaHS dose-dependently increased LC3B-II level in HT-29 cells at 48 h. The expression of Beclin-1 was not altered. (E) Increased autophagic flux was confirmed by co-treating SW1116 cells with NaHS and bafilomycin A_1_. Treatment with bafilomycin A_1_ (10 nmol/L) did not prevent the upregulation of LC3B-II in cells incubated with NaHS (1 mmol/L; 48 h).

To determine if H_2_S induced autophagy in colon epithelial cells, immunofluorescence for LC3^+^ autophagosomes was performed. Results showed that NaHS at the concentration of 1 mmol L^−1^ drastically increased the formation of LC3B^+^ dots or vacuoles in HT-29, SW1116 and HCT116 cells. A modest increase in the formation of LC3B^+^ autophagic vacuoles could be observed as early as 24-h after treatment (**Fig. 3A**). Ultrastructural analysis by electron microscopy also revealed that a 48 h-exposure to H_2_S induced massive vacuolization in HT-29 cells. As shown in **Fig. 3B**, mitochondria encircled by double membrane structures resembling autophagosomes were observed in H_2_S-treated cells. Moreover, a subset of autophagosomes contained lamellar or myelin-like structures. In contrast, control cells exhibited high density of normal mitochondria in the cytoplasm. As the amount of LC3B protein, particularly LC3B-II, has been shown previously to correlate with the extent of autophagy, the effect of H_2_S on LC3B protein expression in colon epithelial cells was studied. Results showed that NaHS at the concentration of 1 mmol L^−1^ significantly induced both LC3B-I and -II protein expression in a time-dependent manner in HT-29, SW1116 and YAMC (**Fig. 3C**). A concentration-dependent induction of LC3B protein expression was also observed in HT-29. However, the expression of beclin-1, another protein involved in macroautophagy, was not significantly altered (**Fig. 3D**). The increase in autophagic flux caused by H_2_S was confirmed by treating SW1116 cells with NaHS and bafilomycin A_1_ (a lysosomotropic agent), alone or in combination. Inhibition of lysosomal function by bafilomycin A_1_ increased the levels of LC3B-II induced by NaHS (**Fig. 3E**), suggesting that H_2_S increased autophagic flux in colon epithelial cell.

### Hydrogen sulfide induced the accumulation of acidic vesicular organelles

**Figure 4:**
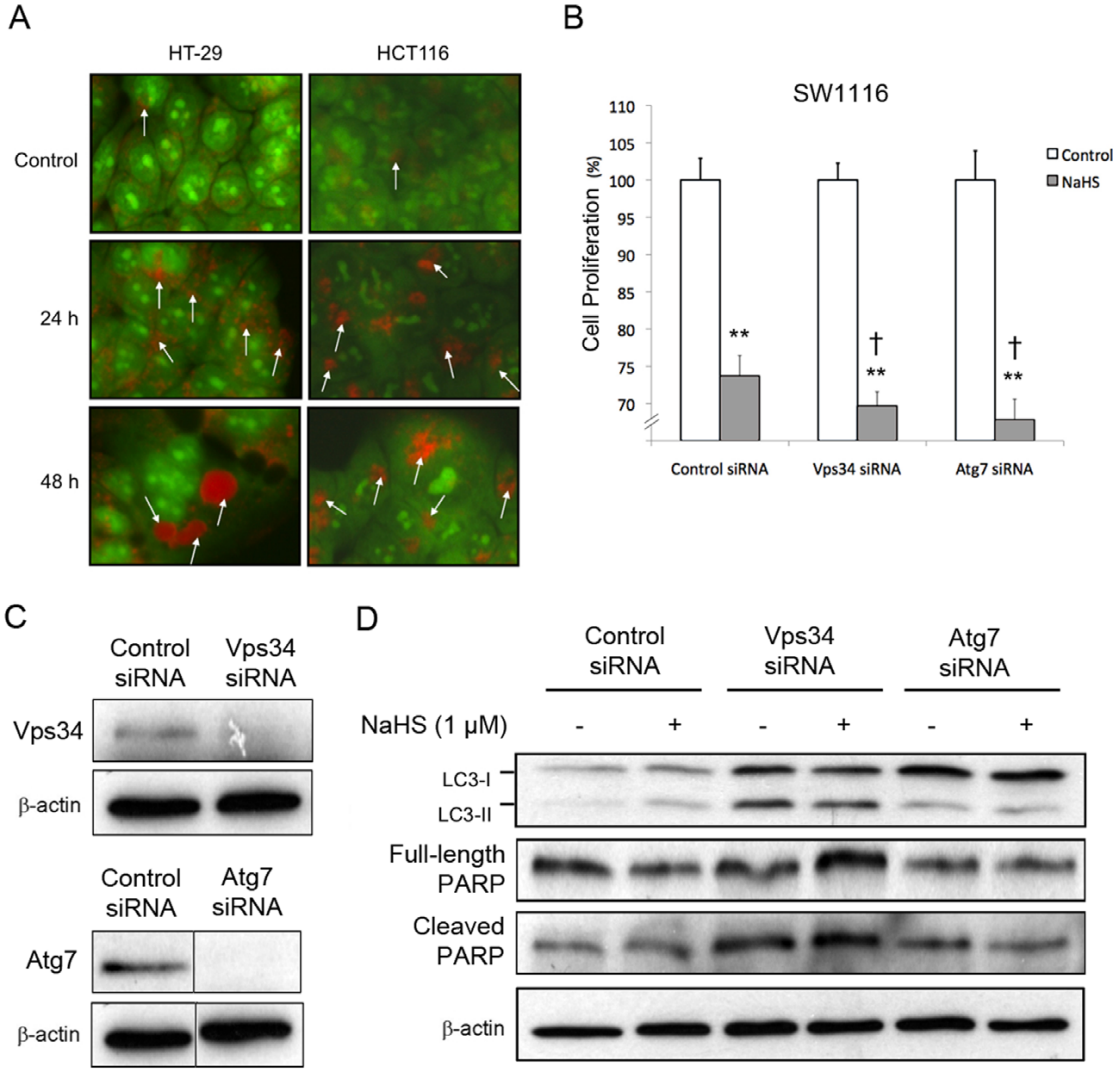
Induction and functional role of autophagy in H_2_S-treated colon epithelial cells. (A) The accumulation of acidic vesicular organelles, which emitted bright red fluorescence (white arrows), induced by 1 mmol/L NaHS was visualized by acridine orange staining. (B) knockdown of Vps34 or Atg7 enhanced the anti-mitogenic effect of 48-h treatment of NaHS (1 mmol/L) in SW1116 cells. (C) The knockdown efficacies of Vps34- and Atg7-siRNAs were confirmed by downregulation of respective targets. (D) Knockdown of Vps34 and Atg7 prevented the upregulation of LC3B-II caused by NaHS (1 mmol/L) but did not alter PARP cleavage. ^**^ *P*<0.01, significantly different from respective control group; † *P*<0.05, significantly different from NaHS group transfected with control siRNA.

Formation of acidic vesicular organelles is an important hallmark of autophagy. For detection of the acidic vesicular organelles, we used the acridine orange staining. Acridine orange emitted bright red fluorescence in acidic vesicles but fluoresced green in cytoplasm and nucleus. Vital staining of HT-29 and HCT116 cells with acridine orange revealed that NaHS enhanced the formation of acidic vesicular organelles in a time-dependent manner (**Fig. 4A**).

### Knockdown of Vps34 or Atg7 enhanced the anti-proliferative effect of Hydrogen sulfide

Depending on cellular context, autophagy may serve as a pro-death or pro-survival mechanism. To determine the functional role of autophagy induced by H_2_S, a RNA interference approach was used to abolish autophagy by targeting Vps34 and Atg7 which are required for the formation of autophagosomes at the early stage. Results show that knockdown of Vps34 or Atg7 significantly enhanced the anti-proliferative effect of NaHS in SW1116 cells (**Fig. 4B**), suggesting that autophagy induced by H_2_S was cytoprotective. The knockdown efficacies of Vps34 and Atg7 siRNAs were confirmed by Western blot (**Fig. 4C**). Knockdown of Vps34 and Atg7 also abolished the increase in LC3B-II levels caused by NaHS (**Fig. 4D**). Since crosstalk may exist between autophagy and apoptotic cell death, we measured PARP cleavage (an apoptosis marker) in SW1116 cells in which autophagy was abolished. Although abolition of autophagy by RNA interference enhanced the anti-proliferative effect of NaHS, knockdown of Vps34 or Atg7 did not affect PARP cleavage in the presence or absence of NaHS (**Fig. 4D**).

### H_2_S induced AMPK and inhibited mTOR signaling

**Figure 5:**
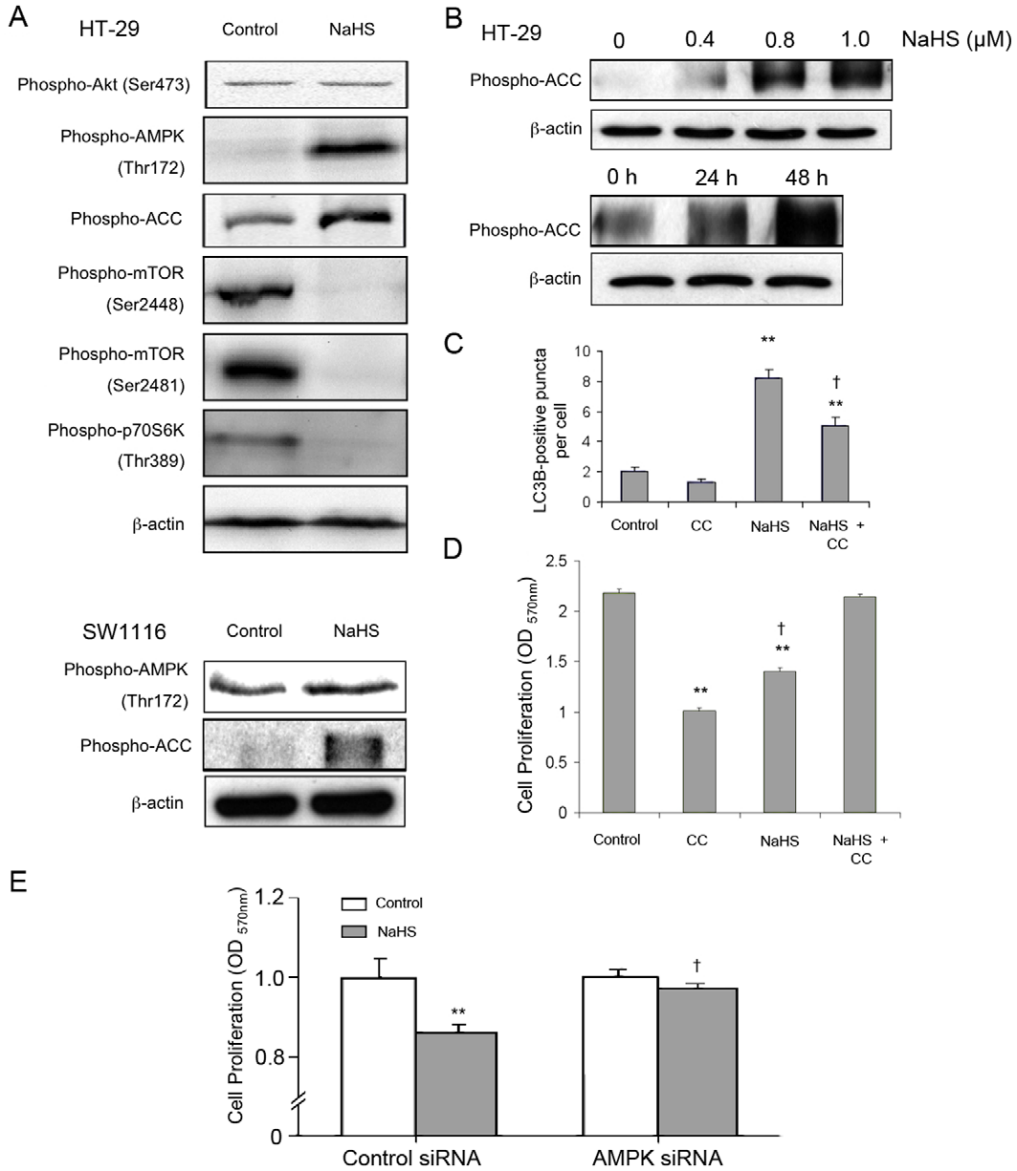
Involvement of AMPK/mTOR pathway. (A) Treating colon epithelial cells with NaHS (1 mmol/L; 24 h) resulted in hyperphosphorylation of AMPK and hypophosphorylation of mTOR and p70 S6K. NaHS (1 mmol/L) also increased phosphorylation of acetyl-CoA-carboxylase (ACC) but did not affect Akt phosphorylation at Ser473. (B) NaHS dose- and time-dependently enhanced ACC phosphorylation in HT-29. (C) The AMPK inhibitor compound c (CC) significantly reduced the number of LC3B^+^ autophagic vacuoles in H_2_S-treated cells. HT-29 cells were treated with compound C (20 mmol/L) for 8 h followed by 1.0 mmol/L NaHS treatment for another 48 h. (D) Compound C (CC) reversed the inhibitory effect of H_2_S on HT-29 cell proliferation. (E) Knockdown of AMPK abolished the inhibitory effect of NaHS (1 mmol/L) on cell proliferation in HCT116. ^**^ *P*<0.01, significantly different from respective control group. † *P*<0.05, significantly different from the NaHS group.

As activation of AMPK is known to play an important role in the induction of autophagy, we measured the phosphorylation of AMPK in HT-29 and SW1116. Results show that H_2_S treatment caused a significant increase of AMPK phosphorylation at Thr-172 in both cell lines. The phosphorylation of AMPK downstrem target acetyl-CoA-carboxylase was also increased by H_2_S in a time- and dose-dependent manner (**Fig. 5A & 5B**). Phospho-AMPK^Thr-172^ is known to phosphorylate and activate TSC2, which inhibits the activation of the downstream targets, such as mTOR and S6 [12]. In this regard, phosphorylation of mTOR and p70 S6 kinase was substantially decreased in H_2_S-treated cells. The phosphorylation of Akt, which has been reported to mediate mTOR phosphorylation, was not affected by H_2_S (**Fig. 5A**). Treating the cells with a specific AMPK inhibitor (compound C) significantly reduced the number of LC3^+^ autophagosomes induced by H_2_S (**Fig. 5C**). Moreover, compound C reversed the anti-proliferative effect of H_2_S in HT-29 (**Fig. 5D**). The importance of AMPK in the regulation of colon epithelial cell proliferation by H_2_S was confirmed by RNA interference. Abolition of AMPK rendered HCT1116 cells insensitive to the anti-proliferative effect of NaHS (**Fig. 5E**).

## Discussion

H_2_S, a gaseous bioactive substance, has recently been implicated in the regulation of cardiovascular, renal and neuronal functions [13], [14], [15]. H_2_S has also been reported to promote gastric ulcer healing in rats [16]. In the colon, where the highest concentration exists, H_2_S regulates chloride secretion, visceral nociceptive processing, and mobility [17], [18], [19]. A recent study also demonstrates that this gaseous compound exerts anti-inflammatory effect in a mouse model of colitis [20]. In relation to carcinogenesis, H_2_S levels are elevated in colon cancer patients. The effect of H_2_S on the physiology of normal and cancerous colon epithelial cells, however, is not clear. Here we show that H_2_S lowers the proliferation in both normal colonocytes and colon cancer cells. H_2_S also induces G_0_/G_1_ arrest and upregulated the expression of CDK inhibitor p21^Cip1^. The anti-mitogenic effect of H_2_S is associated with the induction of autophagy as evidenced by the increased formation of LC3B^+^ autophagic vacuoles and the accumulation of acidic vesicular organelles, as well as accumulation of LC3B-II protein. In addition, autophagy induced by H_2_S is pro-survival. The effects of H_2_S on autophagy occur at concentrations similar to those found in the human colon and coincides with the anti-mitogenic activity. Wound healing assay also demonstrates that H_2_S inhibits cell migration of colon cancer cells. However, the influence of reduced proliferation on wound healing assay cannot be ruled out completely. Moreover, it would be interesting to determine the effects of H_2_S on cell migration in the context of gastric ulcer healing and colitis.

Autophagy is a cellular process that recently becomes a subject of intense investigation because of its role in various pathological states including cancer. While defective autophagy has been suggested to contribute to carcinogenesis, this cellular process promotes cell survival in time of stress and nutrient deprivation [7], [10]. In colon cancer, extensive overexpression of Beclin 1, a protein required for the formation of autophagosomes, has been 21.3% of cases, respectively, in which extensive overexpression is associated with high histological grade, vascular invasion, nodal involvement and poorer overall survival. The absolute levels of several autophagy-related genes, including Atg8, are also higher in tumor tissues in which increased Atg8 expression is correlated with a low grade of differentiation and shortened overall survival [21]. Here we also show that H_2_S concurrently exerts anti-mitogenic and pro-autophagic effects on normal colonocytes and colon cancer cells. Importantly, RNA interference-mediated abolition of autophagy enhances the anti-mitogenic effect of H_2_S. These experimental findings are consistent with the role of autophagy as a pro-survival mechanism in colon cancer and suggest a novel relationship between H_2_S and autophagy.

**Figure 6:**
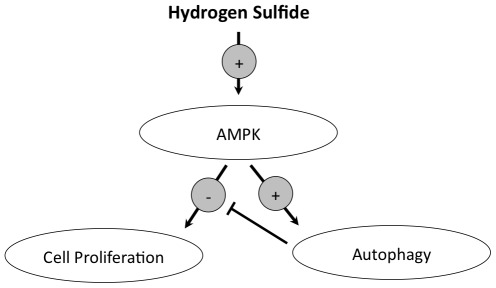
Schematic diagram showing the effects of H_2_S on cell proliferation and autophagy in relation to the AMPK signaling pathway.

Autophagy is known to be induced by AMPK signaling but inhibited by mTOR signaling. For instance, interruption of mTOR function by rapamycin is known to stimulate autophagy, both in mammalian cells and in yeast [11]. Previous studies have also indicated that AMPK inhibits mTOR and induces autophagy in different cell types [22]. In the present study, we demonstrate that H_2_S increases the phosphorylation of AMPK at Thr-172. The induction of AMPK phosphorylation is associated with reduced phosphorylation of mTOR and p70 S6K. Moreover, pharmacological inhibition of AMPK by compound C reverses the effect of H_2_S on cell proliferation and autophagy. The result is also substantiated by RNA interference experiment. These data suggest that the AMPK/mTOR cascade plays a central role in mediating the anti-proliferative effect of H_2_S. Intriguingly, our data suggest that AMPK activation may produce protective autophagy and suppress cell proliferation at the same time in H_2_S-treated colon epithelial cells in which knockdown of autophagy-related genes further enhances the inhibition of cell proliferation (**Fig. 6**). It is speculated that autophagy induced by AMPK activation may countercheck AMPK-mediated suppression of cell proliferation through widespread crosstalk with other metabolic or signaling pathways.

In the colon, H_2_S plays a protective role against the development of colitis by modulating inflammation. H_2_S not only reduces granulocyte infiltration into the colonic tissue but also suppresses the expression of mRNA for several key proinflammatory cytokines [20]. Moreover, several H_2_S-releasing non-steroidal anti-inflammatory drugs have been developed for inflammatory bowel diseases [23]. The risk of developing dysplasia and carcinomas in the colon is known to increase over time in patients with inflammatory bowel diseases. In this respect, H_2_S possesses unique anti-inflammatory properties, rendering this compound potentially beneficial for the treatment of both diseases. However, the inhibitory effect of H_2_S on the proliferation of colonocytes should also be taken into consideration. In conclusion, our study demonstrates that H_2_S lowers proliferation and induces autophagy in colon epithelial cells and such actions depend on the AMPK/mTOR cascade.

## Materials and Methods

### Reagents

All primary antibodies were purchased from Cell Signaling Technology (Beverley, MA, USA) unless otherwise specified. Catalog number of antibody: p15(#4822), p16(#4824), p21 (#2946), β-actin (#4967), LC3B (#2775), Beclin-1 (#3738), p-AMPK (#2535), p-acetyl-CoA-carboxylase (#3661), mTOR (#2983), p-mTOR(Ser2448) (#2971), p-mTOR(Ser2481) (#2974), p-p70S6K (#9234), p-Akt (#4058). Acridine orange and 4′,6-diamidino-2-phenylindole (DAPI) were purchased from Invitrogen (Invitrogen, Carlsbad, CA). All other chemicals and reagents were purchased from Sigma (St. Louis, MO, USA) unless otherwise specified.

### Cell culture and proliferation assay

Young adult mouse colonic (YAMC) cells, conditionally immortalized mouse colonocytes, were originally obtained from R.H. Whitehead, Ludwig Cancer Institute (Melbourne, Australia) and cultured under permissive conditions as previously described [24]. The human colon adenocarcinoma cell lines HT-29, HCT-116 and SW1116 were obtained from the American Type Culture Collection (Manassas, VA) and maintained in RPMI 1640, supplemented with 10% fetal bovine serum, 100 U/mL penicillin and 100 µg/mL streptomycin at 37°C in a humidified atmosphere of 5% CO_2_ and 95% air. Cell proliferation was measured by MTT [3-(4,5-dimethylthiazol-2-yl)-2,5-diphenyltetrazolium bromide] assay. Cells were plated at a density of 5000 cells per well in 96-well plates. After treatment, MTT solution dissolved in the culture medium at the final concentration of 0.5 mmol/L was added to each well and the plates were incubated for another 4 h. Dimethyl sulfoxide was then added to solubilize MTT tetrazolium crystal. Finally, the optical density was determined at 570 nm using a Benchmark Plus microplate reader (Bio-Rad, Hercules, USA). For determination of necrotic cell death, the amount of lactate dehydrogenase released into the supernatant was measured by a cytotoxicity detection kit from Roche Scientific (Indianapolis, IN, USA) according to the manufacturer's recommendation.

### Wound healing assay

A monolayer wound-healing assay was used to measure the effect of H_2_S on the lateral motility of SW1116 cells. In brief, SW1116 cells were seeded onto 24-well plates and cultured in RPMI1640 medium with 10% FBS until confluent. The confluent monolayers were washed twice with PBS and incubated in a serum-free medium. Small linear wounds were created by gently striking a pipette tip across the monolayers. The healing of the wounds through cell migration was assessed by measuring the wound distance.

### Drug treatment

Sodium hydrogen sulfide was used as H_2_S donor. After dissolution, sodium hydrogen sulfide (NaHS) dissociates into Na^+^ and HS^−^. The later partially binds to H^+^ to form undissociated H_2_S, which is lipophilic and freely permeates plasma membranes. H_2_S-releasing medium was prepared by dissolving NaHS into normal growth medium at the indicated concentrations and was changed every 24 h throughout the experiments.

### Cell cycle analysis

HT-29 and SW1116 cells were fixed with ice-cold 70% ethanol in PBS followed by incubation with 50 mg/ml propidium iodide, 3.8 mmol/L sodium citrate, and 0.5 mg/ml RNase A at 4°C for 3 h and analyzed by flow cytometry (Beckman Coulter, Fullerton, CA, USA). The resultant DNA histograms were generated using WinMDI 2.8 software.

### Immunofluorescence for LC3B^+^ autophagic vacuoles

Cells grown on 96-well plates were fixed with 4% (v/v) paraformaldehyde for 30 min and then made permeable with methanol at −20°C for 10 minutes. The cells were then covered with 10% (v/v) goat serum for 30 minutes at room temperature to block nonspecific adsorption of antibodies to the cells. After this procedure, the cells were incubated with primary antibody against LC3B at 4°C overnight. Cells were then probed with Alexa Fluor 488 goat anti-rabbit secondary antibodies and incubated at room temperature for another 2 hours. Fluorescent signals were detected using a fluorescence microscope (Nikon TS100-F). Macroautophagy was quantified by counting the number of LC3B^+^ dots or vacuoles per cells (a minimum of 100 cells per preparation in 2 independent experiments).

### Acridine orange staining for acidic vesicular organelles

Acridine orange was added at a final concentration of 1 mg/ml for a period of 15 min. Pictures were obtained with a fluorescence microscope (Nikon TS100-F) equipped with a 50-W mercury lamp, a 450–490-nm band-pass blue excitation filters, a 505-nm dichroic mirror, a 520-nm long pass-barrier filter, and a digital camera (Nikon DS-5Mc).

### RNA interference

The expression of Class III PI3K Vps34, Atg7 and AMPK was lowered using target-specific siRNA molecules (Vps34—Hs_PI3KC3_5, SI00605822; ATG7—Hs_ATG7L_5, SI02655373; AMPK—Hs_PRKAA2_6,SI02758595) purchased from Qiagen. Two hundred picomoles of gene-specific or control small interference RNA (siRNA) was transfected into cells at 40–60% conuence using Lipofectamine2000 reagent (Invitrogen) according to the manufacturer's instructions.

### Western blot for proteins related to autophagy and cell cycle

Cells were harvested in radioimmunoprecipitation buffer [50 mmol/L Tris–HCl (pH 7.5), 150 mmol/L sodium chloride, 0.5% a-cholate acid, 0.1% SDS, 2 mmol/L EDTA, 1% Triton X-100, and 10% glycerol] containing proteinase and phosphatase inhibitors [1 mmol/L phenylmethylsulfonyl fluoride, 1 mg/ml aprotinin, 1 mg/ml leupeptin, 1 mg/ml pepstatin, 1 mmol/L Na_3_VO_4_, and 1 mmol/L NaF]. Proteins were quantified using protein assay kit (Bio-Rad Laboratories, Hercules, USA). Equal amounts of proteins (50 µg/lane) were resolved by SDS-PAGE, and transferred to Hybond C nitrocellulose membranes (Amersham Corporation, Arlington Heights, IL, USA). The membranes were probed with primary antibodies overnight at 4°C and incubated for 1 h with secondary peroxidase-conjugated antibodies (Invitrogen). Chemiluminescent signals were then developed with Lumiglo reagent (Cell Signaling Technology) and detected by the ChemiDoc XRS gel documentation system (Bio-rad).

### Statistical analysis

Results were expressed as means ± SEM. Statistical analysis was performed with an analysis of variance (ANOVA) followed by the Tukey's t-test. *P* values less than 0.05 were considered statistically significant.
